# Upregulation of Apolipoprotein L6 Improves Tumor Immunotherapy by Inducing Immunogenic Cell Death

**DOI:** 10.3390/biom13030415

**Published:** 2023-02-22

**Authors:** Kecheng Liu, Yutong Chen, Bixiang Li, Yaning Li, Xinyue Liang, Hai Lin, Lisi Luo, Tianliang Chen, Yalan Dai, Wenzheng Pang, Linjuan Zeng

**Affiliations:** The Cancer Center, The Fifth Affiliated Hospital of Sun Yat-sen University, Zhuhai 519000, China

**Keywords:** immunotherapy, immune checkpoint inhibitors, apolipoproteins L, epstein-barr virus infections

## Abstract

In the past few years, immune checkpoint blockade (ICB) therapy has emerged as a breakthrough treatment for cancers and has demonstrated inspiring effects in tumor patients with Epstein-Barr virus (EBV) infection. To allow more patients to benefit from immunotherapy, exploring novel biomarkers based on EBV-related tumors and immunotherapy cohorts was pursued in the present study. The essential biomarkers that may enhance antitumor immunity across EBV-related tumors were identified using the large-scale transcriptomic profiles of EBV-associated tumors and tumor immunotherapy cohorts. The clinical significance of vital genes was evaluated in multiple tumor immunotherapy cohorts. Moreover, the potential function of essential genes in immunotherapy was explored via bioinformatic analyses and verified by qRT-PCR, Western blot analysis, CCK8 assay and flow cytometry. Apolipoprotein L6 (APOL6) was considered the essential biomarker for enhancing antitumor immunity across EBV-positive tumors. The upregulation of APOL6 was correlated with increased response rates and prolonged survival in multiple tumor immunotherapy cohorts. Bioinformatic analyses suggested that APOL6 may enhance tumor immunotherapy by inducing immunogenic cell death. Pancreatic cancer cells transfected with APOL6 overexpression plasmid underwent apoptosis, necroptosis, and pyroptosis with immunogenic features. The biomarker upregulated in EBV-related tumors could further elucidate the drivers of immunotherapy response. The upregulation of APOL6 could improve immunotherapy by triggering immunogenic cell death, thus offering a new target to optimize cancer immunotherapy.

## 1. Introduction

In the past few years, immunotherapy has emerged as a breakthrough treatment for cancers [1]. In particular, immune checkpoint blockade (ICB) therapy has been applied to a growing number of tumor types [2]. However, ICB therapies are only effective in a small subset of tumor patients, and only 60% of the variance in checkpoint inhibitor outcomes can be explained by previously published biomarkers [3]. Therefore, exploring novel biomarkers to optimize the treatment regimen and identify the candidates who would best benefit from ICB therapy is necessary and urgent.

Recent studies have shown that ICB therapy has demonstrated inspiring therapeutic effects in tumor patients with Epstein-Barr virus (EBV) infections [4,5,6]. Compared to EBV-unrelated tumors, the microenvironments of EBV-associated tumors contain higher expression levels of PD-L1 and more proinflammatory immune cells [7,8,9]. These observations led us to speculate that the immune components mediating tumor regression in EBV-related tumors may be a leading determinant of immune responses to ICB therapy. However, the detailed molecular mechanisms of the improved immunotherapy response in EBV-related tumors have not been fully elucidated.

To examine whether genes overexpressed in EBV-related tumors could help further elucidate the drivers of immunotherapy responses, we first identified the essential biomarkers that may enhance antitumor immunity across EBV-related tumors using transcriptomic profiles. The clinical significance of candidate biomarkers was then assessed in tumor immunotherapy cohorts. In addition, the function of candidate biomarkers in cancers was explored via bioinformatic approaches and verification experiments.

## 2. Materials and Methods

### 2.1. Transcriptomic Profile of EBV-Associated Tumors and Tumor Immunotherapy Cohorts

A detailed flowchart of the study design is shown in Figure S1. Epstein-Barr virus (EBV) infection of tumors is defined as the presence of EBV-encoded small RNA (EBER) nuclear signals in tumor cells by in situ hybridization [6,8,10,11]. Gene expression data from classical Hodgkin lymphoma (CHL, GSE13996), diffuse large B-cell lymphoma (DLBCL, GSE38885), plasmablastic lymphoma (PBL, GSE102203), and gastric cancer (GC, GSE51575) were retrieved from the Gene Expression Omnibus database. Several tumor immunotherapy cohorts with publicly available mRNA expression and clinical data were included in the present study. According to the original studies [12,13,14,15], patients with a RECIST response (stable disease lasting more than six months, partial response or complete response) or achieving pathologic complete response were categorized as responders; the other patients were classified as nonresponders. Array and RNA-seq data were analyzed for differential expression using the R packages limma and DESeq2, respectively. Genes with fold changes >1.2 and *p* values < 0.05 were identified as significantly upregulated genes. Notably, only the normalized RNA-seq data of the melanoma cohort [16] were accessed (http://tide.dfci.harvard.edu/download/ (accessed on 15 October 2021)), so the readily available differential genes of DESeq2 analysis were downloaded from the original articles [15].

Venn diagrams indicating the intersection of multiple datasets were constructed using VENNY 2.1 software (https://bioinfogp.cnb.csic.es/tools/venny/ (accessed on 2 June 2022)). To explore the essential biomarkers for enhancing antitumor immunity across EBV-positive tumors, the genes upregulated in both EBV+ tumors and immunotherapy responders were screened using Venn diagrams. The genes overexpressed in EBV+ tumors were obtained from the intersection of multiple EBV-related tumors (EBVaCHL, EBVaDLBCL, EBVaPLB, and EBVaGC). Similarly, the genes overexpressed in immunotherapy responders were defined as the intersection of multiple tumors (melanoma, urothelial cancer, breast cancer, and thymic cancer) receiving immunotherapy.

### 2.2. Association between APOL6 and the Tumor Microenvironment

Immune checkpoint-related gene expression, immune cell infiltration, and tumor cell immunogenicity are three putatively influential factors in tumor immunotherapy. To infer the potential mechanism by which APOL6 may act in cancer immunotherapy, the relationship between the APOL6 expression level and the above three influential factors was evaluated by Spearman’s correlation tests. APOL6 and immune checkpoint-related gene expression profiles (RNA-sequencing expression, level 3) for pancancer were downloaded from The Cancer Genome Atlas (TCGA) database (https://portal.gdc.com (accessed on 15 January 2022)). The count data were converted to transcripts per million (TPM) [17], and the subsequent analyses were performed using TPM if not otherwise stated. TIGIT, SIGLEC15, PDCD1LG2, PDCD1, LAG3, HAVCR2, CTLA4, and CD274 were the immune checkpoint transcripts extracted. To assess the immune cell infiltration in tumors, the CIBERSORT algorithm was used to estimate the proportion of different immune cells in pancancer samples. Finally, the relationship between APOL6 expression and tumor cell immunogenicity (i.e., TMB, MSI and immunogenic cell death burden) was assessed. TMB and MSI data were obtained from the studies published by Vesteinn Thorsson et al. [18] and Russell Bonneville et al. [19], respectively. Gene set variation analysis (GSVA) [20] was performed to estimate immunogenic cell death burden, including necroptosis, pyroptosis, and ferroptosis. Apoptosis, ferroptosis and necroptosis gene sets were obtained from KEGG database, while the pyroptosis gene set was downloaded from the Reactome Database.

### 2.3. Gene Ontology (GO) and Gene Set Enrichment Analysis (GSEA)

The gene expression profile used for GO and GSEA was also accessible from TCGA datasets. For each tumor type, all patients whose APOL6 expression levels were in the bottom 25% and top 25% were classified as having low APOL6 and high APOL6 expression levels, respectively. Differentially expressed genes (DEGs) between the low- and high-APOL6 groups were defined as genes with adjusted *p* values < 0.05 and |log2foldChange| values > 1 from DESeq2 analysis. The DEGs of skin cutaneous melanoma (SKCM) and bladder cancer (BLCA) were subjected to GO enrichment analyses using the R packages clusterProfiler (version 4.2.2) and org.Hs.eg.db (version 3.14.0). The *p* values were adjusted for multiple testing using Benjamini and Hochberg’s false discovery rate (FDR) method; if a *p* value was <0.01 and the corresponding FDR was <0.05, the GO terms were considered statistically significant. With the TPM data of TCGA, GSEA between the low APOL6 and high APOL6 groups was performed via the R packages clusterProfiler (version 4.2.2), org.Hs.eg.db (version 3.14.0) and enrichplot (version 1.14.2). The annotations for the biological pathways were downloaded from the Molecular Signature Database of c2 (c2.cp.kegg.v7.5.1.symbols) and h (h.all.v7.5.1.symbols). Significantly enriched pathways were identified as those with *p* values < 0.05 and FDR values < 0.25 [21].

### 2.4. Cell Counting Kit-8 (CCK8) Assay

To assess the effect of APOL6 on cell viability, CCK8 experiment was performed with a CCK8 kit (Vazyme, Nanjing, China). The pancreatic cancer cell MIA PaCa-2 was obtained from ATCC and was seeded into 96-well plates at a density of 5 × 10^3^ cells/well. The next day, MIA PaCa-2 cells were transfected with APOL6 overexpression plasmid or empty vector plasmid using a liposomal transfection reagent (Yeasen, Shanghai, China). After culturing for 24 h, 48 h, and 72 h, the supernatant was replaced with fresh medium containing 10% CCK8 for additional 1 h. Relative cell viability was calculated as the absorbance of APOL6-transfected cells compared with vector-transfected cells.

### 2.5. Flow Cytometry

Flow cytometry with propidium iodide (PI) staining was conducted to evaluate the effect of APOL6 on cell death. To determine programmed cell death types, MIA PaCa-2 cells transfected with APOL6 overexpression plasmid were preincubated with various cell death inhibitors. Apoptosis inhibitor (Z-VAD-FMK, GC12861), necroptosis inhibitor (Necrostatin-1, GC11008), ferroptosis inhibitor (Ferrostatin-1, GC10380), and pyroptosis inhibitor (VX765, V0024) were dissolved in DMSO [22,23]. In the flow cytometry tests, MIA PaCa-2 cells without any treatment were used as blank controls. Flow cytometry was conducted using CytoFLEX (Beckman Coulter), and the data were analyzed with CytExpert software (Beckman, Brea, CA, USA).

### 2.6. Western Blot Analysis

The total protein of cell samples was extracted using RIPA lysis buffer with protease inhibitors and immediately quantified using a BCA Protein assay kit (Thermo Fisher Scientific, Waltham, MA, USA). A total of 20 µg of protein was separated on a 10% Tris-HCl gel and transferred to a 0.45 μm polyvinylidene difluoride membrane (Millipore, Burlington, MA, USA). The transferred membranes were incubated at 4 °C overnight with the goat anti-APOL6 antibody (ab92273, Abcam, Cambridge, UK). The membranes were then incubated with HRP anti-goat antibody (AS031, Abclonal, Woburn, MA, USA) for one hour.

### 2.7. RNA Extraction, cDNA Synthesis, and qRT-PCR

Total RNA was extracted using an RNA Quick Purification kit (ESscience, Shanghai, China). The RNA concentration was detected according to a 260/280 by an ABI Prism 7900 Sequence Detection System (Applied Biosystems, Waltham, CA, USA). The Hifair^®^ III 1st Strand cDNA Synthesis SuperMix for qPCR (Yeasen, Shanghai, China) was used to synthesize the first-strand cDNA. qRT-PCR assays were performed on an AriaMx Real-Time PCR System (Agilent Technologies, Santa Clara, CA, USA) using Hieff^®^ qPCR SYBR^®^ Green Master Mix (Yeasen, Shanghai, China). The sequences of the qRT-PCR primers are summarized in Table S3. The expression levels of mRNA were quantified relative to GAPDH.

### 2.8. Statistical Analysis

The association between APOL6 and the tumor microenvironment was evaluated by Spearman correlation analysis. A Spearman correlation coefficient >0.8 was considered to reflect high correlation; a value of 0.5–0.8 conferred moderate correlation, and a value <0.5 indicated that the factors were not well correlated. Logistic regression analyses were undertaken to assess the association of APOL6 expression status (i.e., high vs. low) with immunotherapy response. Stratified and interaction analyses were performed according to the treatment regimens (i.e., with immune checkpoint inhibitors vs. without immune checkpoint inhibitors). To examine the associations of APOL6 expression with patient survival, Kaplan–Meier estimates, restricted cubic spline curves [24,25], and multivariable-adjusted Cox proportional hazards regression analyses were conducted. The confounding variables to be included in the multivariate logistic regression and Cox regression analyses were selected considering the results of the univariate analyses and their clinical importance. *p* values < 0.05 were considered statistically significant. All the above analyses were performed using the R statistical software (v4.1.0).

## 3. Results

### 3.1. Exploring the Critical Genes Enhancing Antitumor Immunity across EBV-Positive Tumors

**Figure 1:**
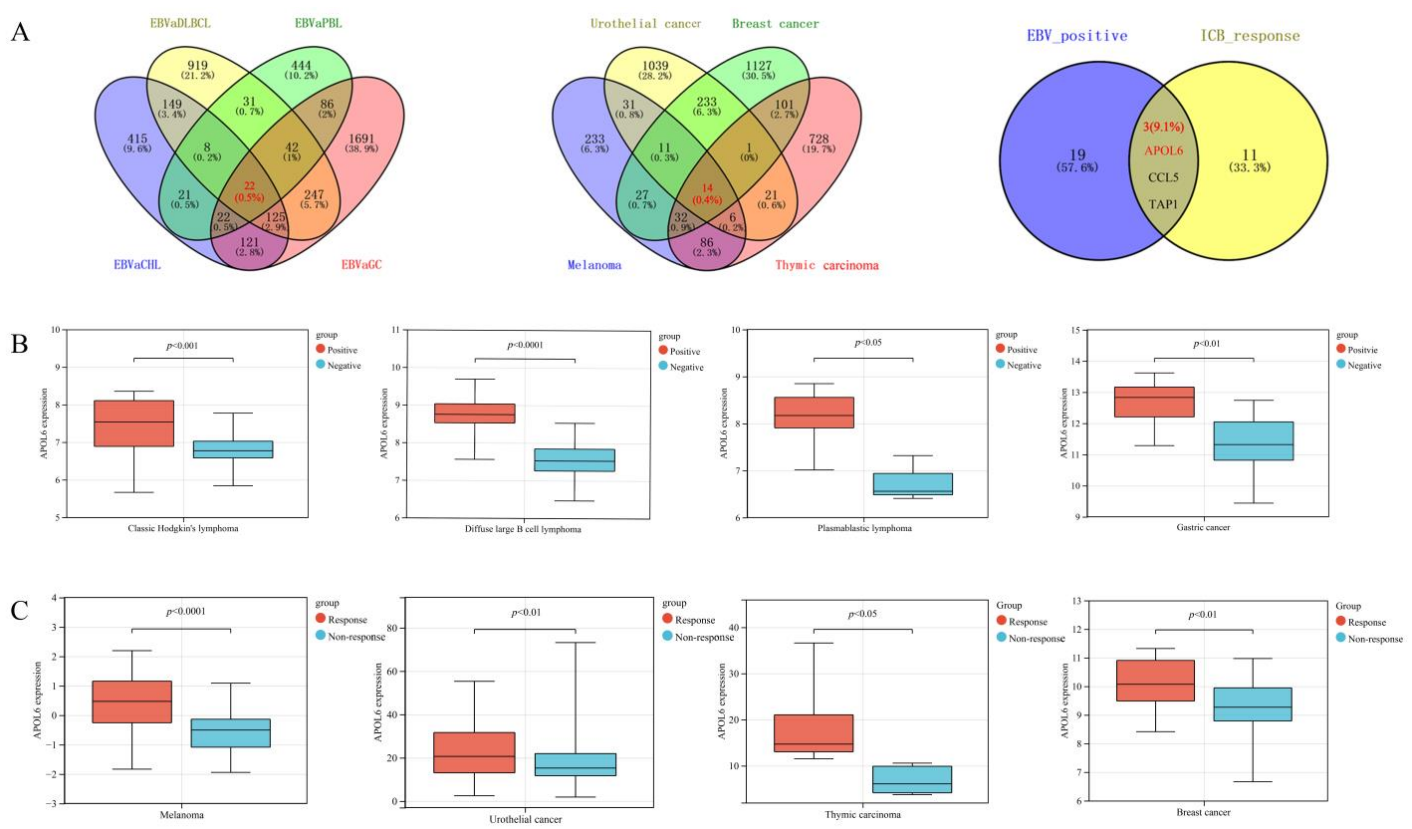
APOL6 was upregulated in EBV+ tumors and in patients responding to immunotherapy. (**A**) Venn diagram displaying the intersection of multiple datasets was constructed to identify the genes upregulated in both EBV+ tumors and immunotherapy responders. The detailed upregulated genes are listed in Table S9. (**B**) APOL6 was upregulated in EBV+ tumors. (**C**) APOL6 was upregulated in the patients responding to immunotherapy. The comparisons were performed with the Wilcoxon rank sum test.

The derived Venn diagram (Figure 1A) shows that 22 genes were upregulated across the EBV+ tumors (CHL, DLBCL, PLB, and GC). Moreover, 14 genes were overexpressed across patients (melanoma, urothelial cancer, breast cancer, and thymic cancer) responding to immunotherapy. Of the genes significantly upregulated in EBV+ tumors, three genes (i.e., APOL6, CCL5 and TAP1) were also significantly highly expressed in responders. Finally, APOL6, CCL5 and TAP1 were considered the key biomarkers to enhance antitumor immunity across EBV-positive tumors. CCL5 and TAP1 have been proven to improve antitumor immunity by attracting CD8+ T-cell infiltration [26,27] and augmenting MHC-I antigen presentation [28], respectively. APOL6 was significantly upregulated in both EBV+ tumors and patients responding to immunotherapy (all *p* values < 0.05, as shown in Figure 1B,C). However, the influence of APOL6 on tumor immunotherapy remains uncharacterized.

### 3.2. Upregulation of APOL6 Correlated with Better Immunotherapy Response and Prognosis

**Figure 2:**
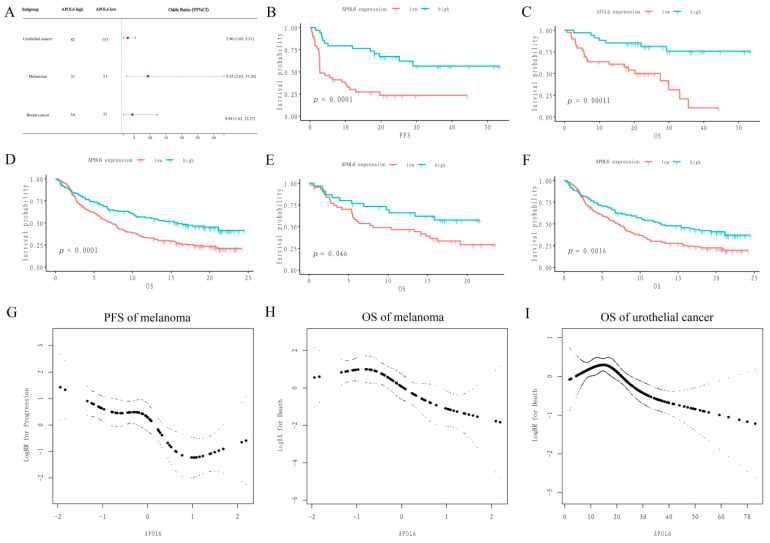
The upregulation of APOL6 correlated with better immunotherapy response and prognosis across multiple tumor types. (**A**) Forest plot of the odds ratios for the response to immunotherapy across multiple tumor types. (**B**,**C**) Kaplan-Meier curves of PFS (**B**) and OS (**C**) in melanoma patients receiving anti-PD1 therapy or a combination of anti-PD1 and anti-CTLA4 immunotherapy. (**D**–**F**) Kaplan–Meier curves of OS in all urothelial cancer patients (**D**), cisplatin-refractory urothelial cancer (**E**), and cisplatin-ineligible urothelial cancer patients (**F**) from the IMvigor210 cohort. (**G**–**I**) Fit curves obtained from restricted cubic smoothing demonstrate the relationship between APOL6 expression and the risk of progression or death. The thick lines and thin lines represent the estimated values and corresponding 95% confidence intervals, respectively.

The effect of APOL6 on tumor immunotherapy was evaluated in several immunotherapy cohorts. Univariate logistic regression indicated that the upregulation of APOL6 was associated with improved response rates in melanoma, urothelial cancer and breast cancer (the ORs (95%CI) were 9.35 (2.63, 33.26), 2.96 (1.65, 5.31) and 4.44 (1.61, 12.27), respectively, as shown in Figure 2A, Table 1 and Table S1). After adjusting for clinical covariates, the upregulation of APOL6 remained a protective factor for improved response (the ORs (95%CI) were 12.46 (3.11, 50.01) and 2.08 (0.97, 4.46) for melanoma and urothelial cancer, respectively, as shown in Table 1 and Table S1). To exclude the possibility that APOL6-upregulated tumors have a generally better response irrespective of immunotherapy, a test was performed to explore any interactions with the treatment regimen. As shown in Table 2, breast cancer patients treated with chemotherapy (i.e., paclitaxel) showed no difference in ORs between the low- and high-APOL6 groups. Although the formal treatment regimen × APOL6 expression interaction analysis was not statistically significant (*p* interaction = 0.12) due to the small sample size, it was evident that the influence of APOL6 expression on efficacy differed between the treatment regimens with and without immune checkpoint inhibitors. These phenomena suggested that APOL6 may be a specific target for improving immunotherapy response in tumors.

The Kaplan–Meier survival curves showed that the upregulation of APOL6 was associated with prolonged survival in melanoma (*p* values < 0.001, Figure 2B,C) and urothelial cancer (*p* values < 0.05, Figure 2D–F). Furthermore, multivariable regression analyses confirmed the independent protective effect of APOL6 overexpression (Table 3 and Table S2). Interestingly, as shown in Figure 2G,H, restricted cubic spline curves showed a nonlinear association between APOL6 expression and the risks of progression and death in melanoma (*p* values for nonlinear trend were <0.001 and 0.012, respectively). A nonlinear relationship also appeared in urothelial cancer (the *p*-value derived for the nonlinear trend was <0.001, Figure 2I). Specifically, the upregulation of APOL6 resulted in a trend that the risks of progression and death first remained relatively constant (plateau phase) and then decreased. The dose–response relationships of APOL6 and risks of progression/death raised the possibility that increased APOL6 expression in tumor tissues might improve prognosis in patients receiving immunotherapy.

Taken together, these results suggest that the upregulation of APOL6 may prolong patient survival by improving the immunotherapy response in multiple tumor types.

### 3.3. Role of APOL6 in Improving Cancer Immunotherapy Based on Bioinformatics Analyses

**Figure 3:**
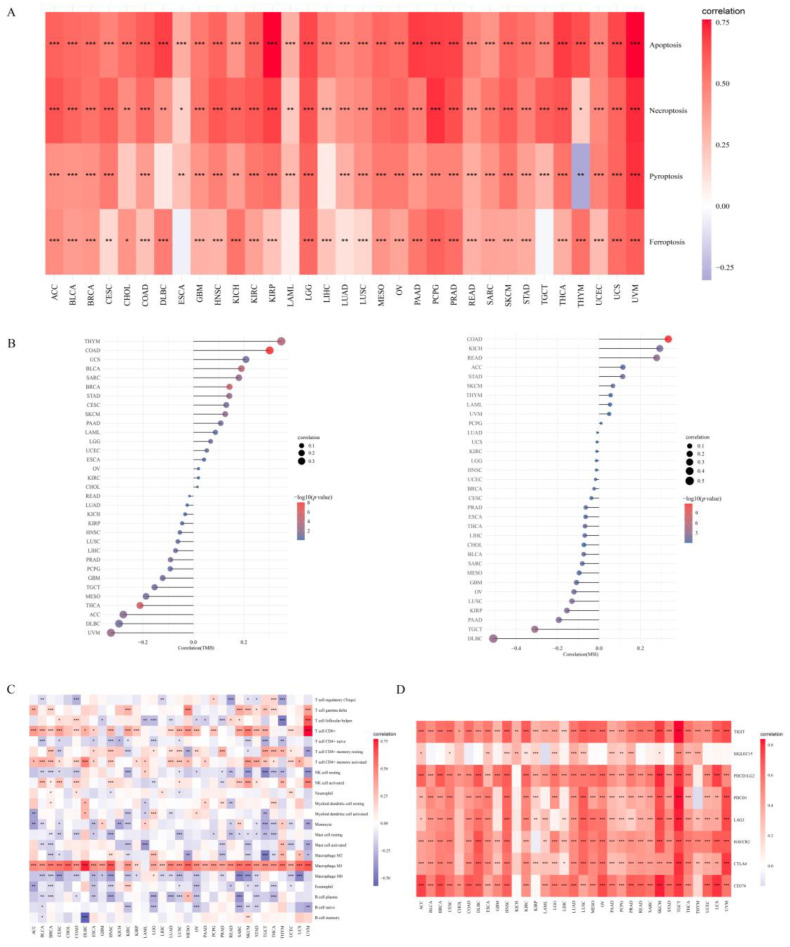
Association of APOL6 levels with cell death burden, immune cell inflation, immune checkpoints, tumor mutation burden (TMB) and microsatellite instability (MSI). (**A**,**C**,**D**) Heatmap of the cell death score, CIBERSORT immune score, immune checkpoint-related gene expression and APOL6 expression in multiple tumor tissues. Each box in the figure represents the Spearman correlation analysis between the APOL6 expression and cell death score, CIBERSORT immune score and immune checkpoint-related gene expression in corresponding tumors. (**B**) Spearman correlation analysis of TMB, MSI and APOL6 gene expression. * *p* < 0.05, ** *p* < 0.01, *** *p* < 0.001.

To investigate the mechanism by which APOL6 enhances anticancer immunotherapy, bioinformatics analyses were performed to clarify the role of APOL6 in the tumor microenvironment. As shown in Figure 3A, APOL6 expression was positively correlated with necroptosis score, pyroptosis score, and ferroptosis score across pancancer. Necroptosis, pyroptosis, and ferroptosis represent the recently discovered immunogenic cell death forms. The induction of necroptosis, pyroptosis, and ferroptosis could reverse ‘cold’ non-T-cell-inflamed tumors into ‘hot’ inflamed tumors for improving ICB therapy outcomes [29,30]. Except for DLBCL, APOL6 expression was weakly associated with TMB or MSI in all other cancers (the absolute values of the correlation coefficients were <0.5, Figure 3B). Figure 3C,D exhibited that APOL6 expression showed a moderately or strongly positive correlation with proinflammatory immune cells (e.g., CD8+ T cells and M1 macrophage) and classical immune checkpoint-related gene expression across most cancers (correlation coefficient > 0.5). Collectively, the upregulation of APOL6 may enhance ICB therapy by promoting immunogenic cell death in cancers.

**Figure 4:**
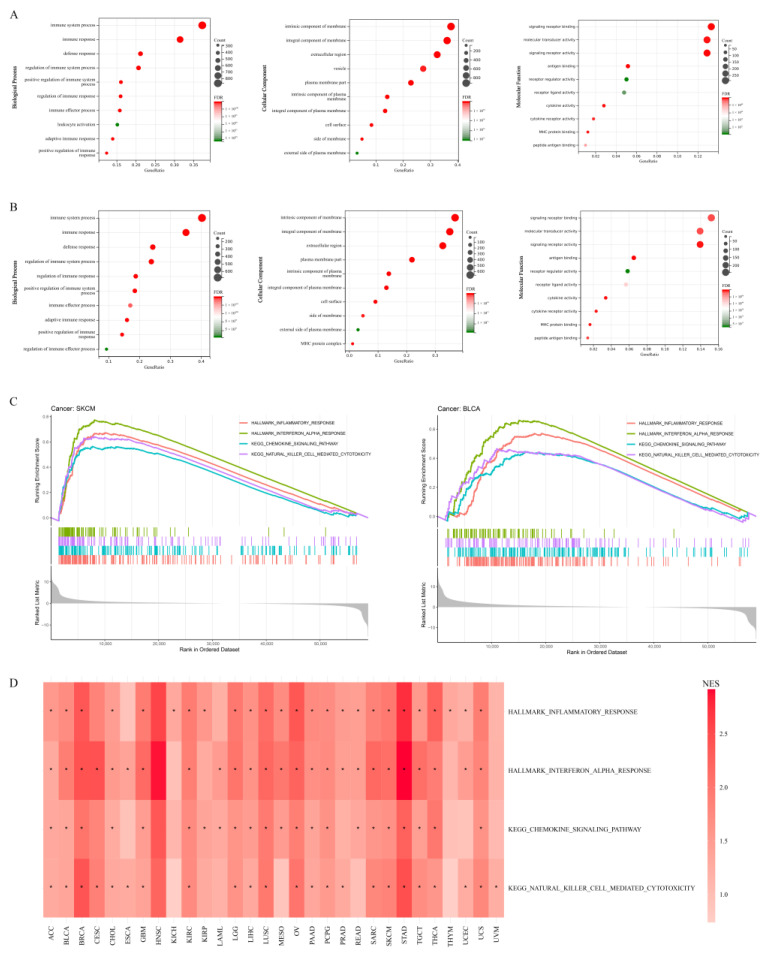
Functional enrichment analysis of APOL6 in cancers. (**A**,**B**) Gene ontology (GO) analysis: top ten terms of GO function analysis in melanoma (**A**) and urothelial cancer (**B**) patients are shown. (**C**) Gene set enrichment analysis (GSEA) of the KEGG pathway and HALLMARK pathway in melanoma and urothelial cancer: Four pathways associated with the immune response to immunogenic cell death are shown. (**D**) Normalized enrichment scores (NESs) of the four pathways from GSEA across pancancer. * *p* < 0.05.

To further explore the possible mechanisms of APOL6 in cancers, GO and GSEA analyses were performed. According to the previous analysis, the upregulation of APOL6 was an independent protective factor for improved response and prognosis in melanoma and urothelial cancer (including BLCA). As examples, the GO and GSEA analyses of melanoma and BLCA were displayed. As a regulator of lipid metabolism, APOL6 affected metabolic processes such as regulation of intestinal lipid absorption (GO: 1904729), positive regulation of fatty acid transport (GO: 2000193) and lipid binding (GO: 0008289) in melanoma (Table S4). Interestingly, the top ten enriched GO terms for biological processes were all related to immune regulation (Figure 4A). Likewise, many of the above enriched GO terms were also enriched in bladder cancer (Figure 4B, Table S5).

Consistent with GO analysis, GSEA revealed a specific enrichment of pathways (e.g., HALLMARK_INFLAMMATORY_RESPONSE) involved in the immune response to immunogenic cell death (Figure 4C, Tables S6 and S7). Cancer cells undergoing immunogenic cell death have been confirmed to release self-dsRNA into the extracellular space. The self-dsRNA initiates type I interferon response consisting of IFNα, IFNβ, and CXCL10 by binding TLR3 on cancer cells in a paracrine manner. This type I IFN response could recruit proinflammatory immune cells (e.g., CD8+ T cells and NK cells), thereby enhancing efficient antitumor immunity [31,32]. These results further support that the upregulation of APOL6 plays a vital role in immunogenic tumor cell death. More importantly, the pathways associated with immunogenic cell death were also significantly enriched in most tumors (Figure 4D, Table S8), supporting that APOL6 may be a pancancer biomarker.

### 3.4. APOL6 Induced Immunogenic Tumor Cell Death In Vitro

**Figure 5:**
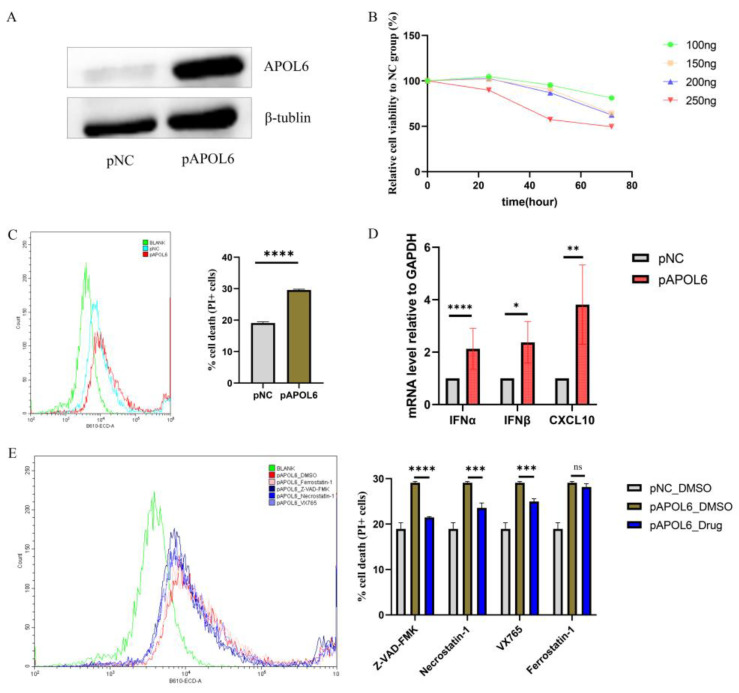
APOL6 induced immunogenic cell death in vitro. (**A**) Immunoblot analysis of FLAG-tagged APOL6 expression in transfected MIA PaCa-2 cells. (**B**) Cell viability of APOL6-transfected MIA PaCa-2 cells was determined using CCK8 assay. MIA PaCa-2 cells were seeded in 96-well plates and transfected with various doses of APOL6 overexpression plasmid for the indicated time-points. The APOL6 protein amount in MIA PaCa-2 cells transfected with APOL6 expression plasmids at different doses was shown in Figure S2. (**C**) Cell death of APOL6-transfected MIA PaCa-2 cells was detected with PI staining by flow cytometry. (**D**) The expression of cytokines and chemokines involved in type I interferon response was measured by qRT-PCR. (**E**) APOL6-transfected MIA PaCa-2 cells were treated with Z-VAD-FMK (10 µM) [23], necrostatin-1 (10 µM) [23], VX765 (10 µM) [22] and ferrostatin-1 (2 µM) [23] for 72 h, and cell death was evaluated with PI staining by flow cytometry. Except where noted, FLAG-tagged APOL6 expression plasmids were transfected in 6-well plates at doses of 5000 ng. The *p* values were calculated using the *t*-test. NC, negative control. * *p* < 0.05, ** *p* < 0.01, *** *p* < 0.001, **** *p* < 0.0001.

Bioinformatic analyses predicted that upregulation of APOL6 may cause multiple forms of cell death in pancreatic cancer (shown in Figure 3A and Figure 4D). To directly determine whether APOL6 could induce immunogenic tumor cell death, APOL6 was upregulated in the pancreatic cancer MIA PaCa-2 cells by transfecting the APOL6 overexpression plasmid (Figure 5A). The CCK8 assay indicated that the upregulation of APOL6 inhibited cell viability through the dose-dependent and time-dependent manner (Figure 5B). Consistent with the decreased cell viability, the flow cytometry results showed that the upregulation of APOL6 resulted in an increased proportion of dead cells (Figure 5C, *p* value < 0.0001).

Cancer cells undergoing immunogenic cell death have been confirmed to initiate type I interferon response, which would enhance efficient antitumor immunity [31,32]. As expected, IFNα, IFNβ, and CXCL10 expression increases in APOL6-transfected MIA PaCa-2 cells, suggesting that upregulating APOL6 in pancreatic cells could induce immunogenic effects (Figure 5D, all *p* value < 0.05). APOL6 induces apoptosis in cancer cells [33], but apoptotic cells are considered nonimmunogenic [34,35]. To explain the immunogenicity observed, the effects of various cell death inhibitors on APOL6-induced cell death were investigated. As shown in Figure 5E, the inhibitors of apoptosis (Z-VAD-FMK), necroptosis (Necrostatin-1) and pyroptosis (VX765) partially rescue APOL6-induced cell death in MIA PaCa-2 cells, whereas ferroptosis inhibitor (ferrostatin-1) does not. Therefore, the upregulation of APOL6 could induce multiple cell death phenotypes, including immunogenic cell death, thereby improving antitumor immunotherapy.

## 4. Discussion

Although immune checkpoint inhibitors have been widely used to treat cancers, serious hurdles remain, such as the low response rates to these inhibitors in most cancers [36,37]. To allow more tumor patients to benefit from immunotherapy, a more comprehensive understanding of ICB therapy must be achieved through further research. EBV-related tumors have been proven to be more eligible for immune checkpoint blockading than EBV-unrelated tumors, and the EBV infection status could thus help identify the tumor patients most likely to benefit from immunotherapy [38]. In the present study, we explored whether the biomarkers upregulated in EBV-related tumors could further clarify the drivers of ICB therapy response. Our results indicate that APOL6, CCL5 and TAP1 were upregulated in EBV+ tumors and immunotherapy responders. CCL5 and TAP1 have been shown to play critical roles in enhancing antitumor immunity by attracting CD8+ T-cell infiltration [26,27] and augmenting MHC-I antigen presentation [28]. The function of APOL6 in tumor immunotherapy remains unknown, but was further characterized in this study. Analysis of multiple clinical cohorts revealed that the upregulation of APOL6 may increase the response rate and improve patient survival. Bioinformatic analyses suggested that APOL6 may induce immunogenic cell death, and this finding was confirmed by in vitro experiments. The novel biomarker APOL6 offers a new approach to optimizing cancer immunotherapy.

Recently, Litchfield et al. summarized T-cell-intrinsic and tumor-intrinsic mechanisms of sensitization to ICB therapy [3]. They reported that more than 40% of the factors determining ICB therapy outcome remain to be discovered or lie outside the transcriptome/exome. In this context, Litchfield et al. further explored novel biomarkers based on clonal neoantigen-reactive T cells using single-cell RNA sequencing. However, the potential biomarkers beyond clonal neoantigen-reactive T cells were not considered. As immune “hot” tumors, EBV-related tumors show a generally favorable response to ICB therapy [4,6,39]. Our exploratory research based on EBV-related tumors and ICB therapy cohorts is an important complement to previous studies.

APOL6 is a member of the apolipoprotein L gene family. As a regulator of lipid metabolism, the upregulation of APOL6 could promote the differentiation of 3T3-L1 preadipocytes and adipogenesis [40]. Besides, APOL6 was identified as one novel BH3-only pro-apoptotic protein and overexpression of APOL6 could induce apoptosis in cancer cells [33]. Increasing evidence proved that complicated crosstalk exists among programmed cell death pathways; apoptosis, necroptosis, and pyroptosis could simultaneously occur during infection, sterile inflammation and cancer [41]. In this study, bioinformatics analysis showed that APOL6 expression was positively associated with multiple programmed cell death scores, suggesting that APOL6 may regulate multiple programmed cell death. As expected, our data confirmed that the upregulation of APOL6 promoted necroptosis and pyroptosis in pancreatic cancer. The induction of necroptosis and pyroptosis could augment anti-tumor immune effects, which explains why APOL6 was upregulated in immunotherapy responders. Unexpectedly, APOL6 was significantly upregulated in EBV+ tumors. The role of APOL6 in EBV infection has not yet been reported, but APOL6 is known to help control zika virus and human immunodeficiency virus [42,43]. Despite the lack of direct literature support, it is reasonable that APOL6 is overexpressed in EBV+ tumors.

Various cells with viral infections are capable of producing type I interferon response [44,45], which attracts abundant immune cell infiltration. Not surprisingly, EBV-related tumors are characterized as immune “hot” tumors with a strong adaptive immune response [46,47,48]. However, increasing immune cell infiltration in tumors by artificial EBV infections would not be ethical. In the present study, we found that APOL6-mediated immunogenic cell death could account for the abundant immune cell infiltration in EBV-related tumors. Furthermore, APOL6 was predicted to participate in metabolic processes, including in the regulation of intestinal lipid absorption, suggesting that APOL6 may be a promising target for diet therapy. In line with this, it has been reported that an arachidonic acid diet could upregulate APOL6 in fish [49]. Therefore, the findings of the present study not only provide a more comprehensive understanding of the immunotherapy response but also offer a new approach to optimize cancer immunotherapy.

This study has several limitations. Firstly, our bioinformatics data indicated that APOL6 might be a pancancer biomarker for ICB therapies. The results should be further validated using other cell lines more related to EBV-related tumors. Moreover, the clinical significance of APOL6 was only evaluated in melanoma, urothelial cancer and breast cancer cohorts. Further clinical studies are required to evaluate its clinical value in other cancers receiving ICIs therapy alone and in combination with other therapies. Secondly, the raw count data were used for differential gene expression analyses by the DESeq2 package to ensure the homogeneity of gene expression profiles. However, only the normalized RNA-seq data of the melanoma cohort could be accessed. Fortunately, the readily available differential genes of DESeq2 analyses could be obtained from supplementary Tables S3 and S5 of that paper [15]. Thirdly, the proof of immunogenic cell death should be further carried out by the activation of dendritic cells via co-culture experiments.

## 5. Conclusions

In summary, the biomarkers upregulated in EBV-related tumors could further clarify the drivers of ICB therapy response. The upregulation of APOL6 improved ICB therapy by inducing immunogenic cell death, thus providing a new therapeutic target for enhancing tumor immunotherapy.

## Figures and Tables

**Table 1:** Univariate and multivariate analyses of factors associated with response to immunotherapy in the melanoma cohort.

	Statistics	Crude Analysis		Adjust Analysis	
		OR (95%CI)	*p* Value ^a^	OR (95%CI)	*p* Value ^a^
**APOL6 expression** ^b^					
Low	31 (50.0%)	1		1	
High	31 (50.0%)	9.35 (2.63, 33.26)	<0.01	12.46 (3.11, 50.01)	<0.01 ^c^
**Treatment regimen**					
PD1	35 (56.5%)	1			
PD1 plus CTLA4	27 (43.5%)	2.95 (0.96, 9.08)	0.06		
**Biopsy Site**					
Subcutaneous	38 (61.3%)	1			
Lymph node	17 (27.4%)	0.74 (0.23, 2.41)	0.62		
Others	7 (11.3%)	1.30 (0.22, 7.64)	0.77		
**Age**	62.3 ± 14.1	1.00 (0.96, 1.04)	0.95		
**Sex**					
Male	39 (62.9%)	1			
Female	23 (37.1%)	1.43 (0.48, 4.28)	0.52		

^a^ *p* values are derived from univariate and multivariate logistic regression models. ^b^ Low and high, respectively, indicate lower than or higher than the mean expression level of APOL6 among the whole melanoma cohort. ^c^ Adjusted for treatment regimen.

**Table 2:** Effects of APOL6 expression on pathologic complete response according to treatment regimen in HER2-negative stage II/III breast cancer.

Treatment Regimen	APOL6 Expression ^a^	Numbers of Patients	OR (95%CI) ^b^
**Paclitaxel**	Low	17	Ref.
	High	17	1.00 (0.24, 4.08)
**Durvalumab, Olaparib and Paclitaxel**	Low	36	0.61 (0.18, 2.13)
	High	35	2.44 (0.74, 8.11)
***p*** **value for interaction**			0.12

^a^ APOL6 expression: low and high, respectively, indicate lower than or higher than the mean expression level of APOL6 among the whole breast cancer cohort. ^b^ ORs were derived from univariate logistic regression models.

**Table 3:** Univariate and multivariate analyses of factors associated with patient survival in the melanoma cohort receiving immunotherapy.

	Statistics	Crude Analysis		Adjust Analysis	
		HR (95%CI)	*p* Value ^a^	HR (95%CI)	*p* Value ^a^
**PFS status**					
**APOL6 expression** ^b^					
Low	39 (53.4%)	1		1	
High	34 (46.6%)	0.27 (0.14, 0.54)	<0.01	0.26 (0.13, 0.51)	<0.01 ^c^
**Treatment regimen**					
PD1	41 (56.2%)	1			
PD1 plus CTLA4	32 (43.8%)	0.52 (0.27, 1.01)	0.05		
**Biopsy Site**					
Subcutaneous	47 (64.4%)	1			
Lymph node	19 (26.0%)	0.84 (0.41, 1.71)	0.63		
Others	7 (9.6%)	0.40 (0.10, 1.68)	0.21		
**Age**	61.6 ± 13.8	1.00 (0.97, 1.02)	0.71		
**Sex**					
Male	47 (64.4%)	1			
Female	26 (35.6%)	0.73 (0.38, 1.40)	0.34		
**OS status**					
**APOL6 expression** ^b^					
Low	39 (53.4%)	1		1	
High	34 (46.6%)	0.21 (0.09, 0.49)	<0.01	0.20 (0.08, 0.48)	<0.01 ^c^
**Treatment regimen**					
PD1	41 (56.2%)	1			
PD1 plus CTLA4	32 (43.8%)	0.28(0.10, 0.74)	0.01		
**Biopsy Site**					
Subcutaneous	47 (64.4%)	1			
Lymph node	19 (26.0%)	0.90 (0.38, 2.11)	0.80		
Others	7 (9.6%)	0.32 (0.04, 2.38)	0.27		
**Age**	61.6 ± 13.8	1.01 (0.98, 1.04)	0.51		
**Sex**					
Male	47 (64.4%)	1			
Female	26 (35.6%)	0.52 (0.22, 1.23)	0.14		

^a^ *p* values are derived from univariate and multivariate Cox proportional hazards regression model. ^b^ APOL6 expression: low and high, respectively, indicate lower than or higher than the mean expression level of APOL6 among the whole melanoma cohort. ^c^ Adjusted for treatment regimen.

## Data Availability

The data and materials supporting our results could be available upon reasonable request.

## Supplementary Materials

The following supporting information can be downloaded at: https://www.mdpi.com/article/10.3390/biom13030415/s1, Table S1: Univariate and multivariate analyses of factors associated with response to immunotherapy in the urothelial cancer cohort; Table S2: Univariate and multivariate analyses of factors associated with overall survival in the urothelial cancer cohort receiving immunotherapy; Table S3: Sequences of qRT-PCR primers used in the present study; Table S4: GO analysis of significantly differentially expressed genes in melanoma; Table S5: GO analysis of significantly differentially expressed genes in bladder cancer; Table S6: GSEA enrichment pathways in melanoma; Table S7: GSEA enrichment pathways in bladder cancer; Table S8: Detailed NESs of the pathways associated with the immune response to immunogenic cell death across pancancer; Table S9: Detailed genes overexpressed in EBV+ tumors and immunotherapy responders; Figure S1: Study flowchart; Figure S2: Immunoblot analysis of APOL6 protein amount in MIA PaCa-2 cells transfected with FLAG-tagged APOL6 expression plasmids at different doses.
