# Supplementary material for: Upregulation of Apolipoprotein L6 Improves Tumor Immunotherapy by Inducing Immunogenic Cell Death
Source: Biomolecules. 2023 Feb 22;13(3):415. doi: 10.3390/biom13030415 (PMC10046184; doi:10.3390/biom13030415)
Supplement: Supplementary file 1 [file biomolecules-13-00415-s001.zip › Supplementary Materials, Figures S1 and S2, Tables S1 and S2.pdf]

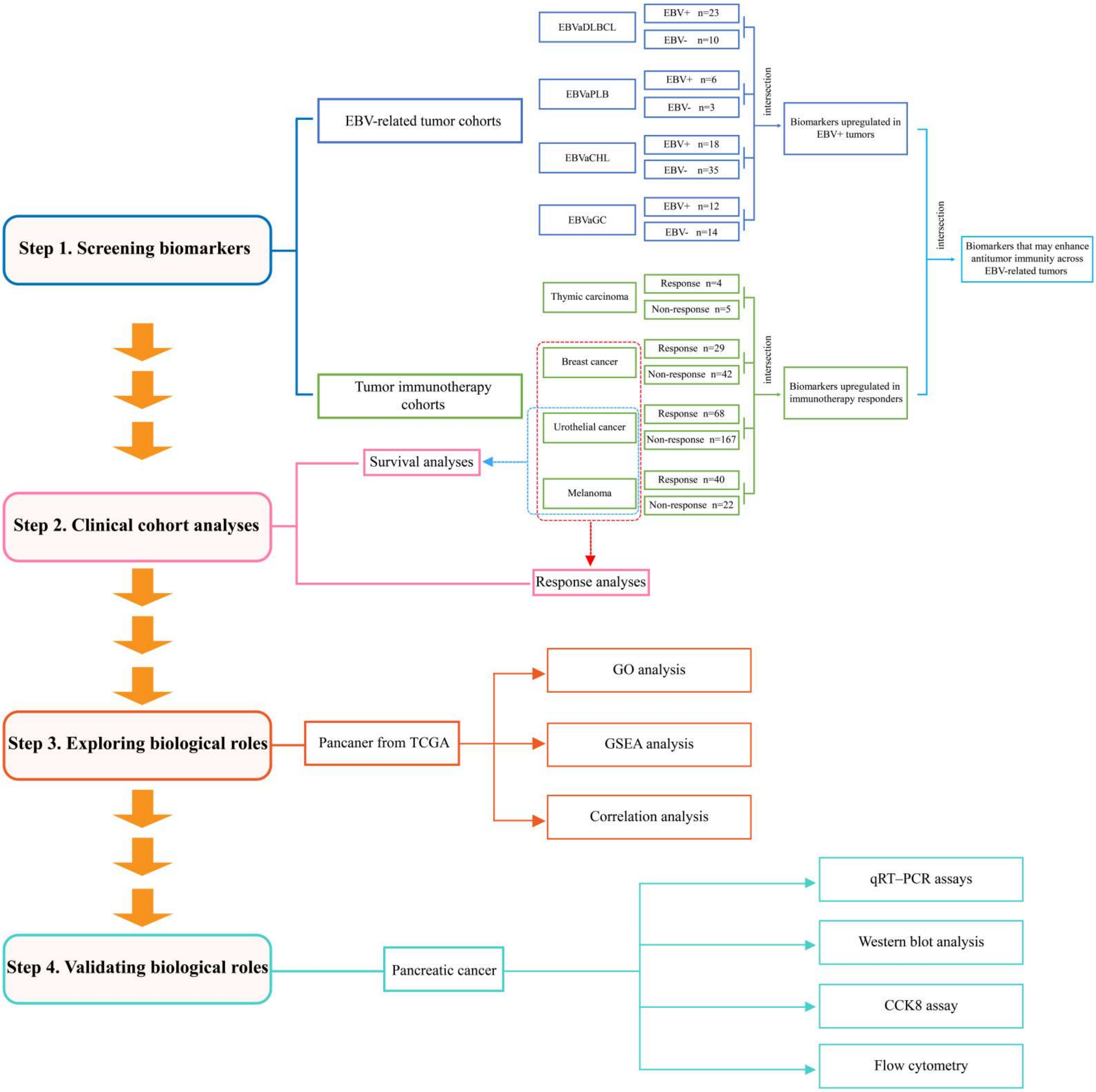

Figure S1. Study flowchart. Step 1 Screening the critical biomarkers enhancing antitumor immunity across EBV-positive tumors. **Step 2** Assessing the clinical values of APOL6 in pan-cancer based on immunotherapy cohorts. **Step 3** Exploring the biological roles of APOL6 in pan-cancer by bioinformatics analysis based on the TCGA database. **Step 4** Validating the biological roles of APOL6 in pancreatic cancer in vitro.

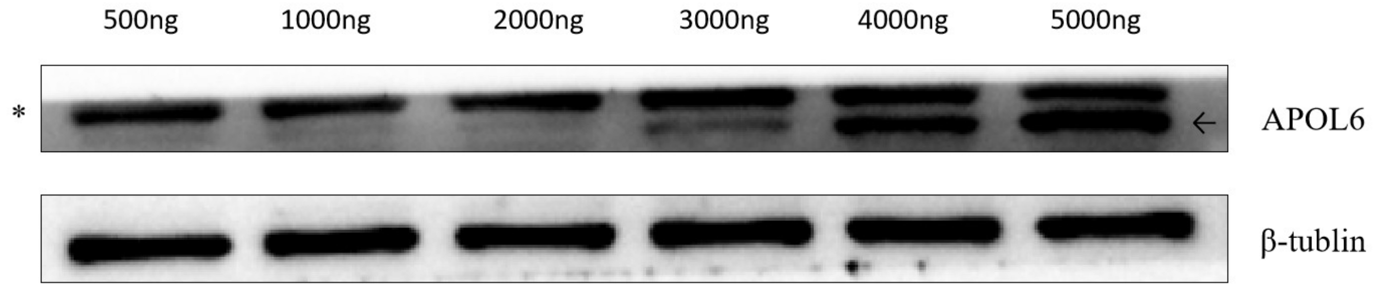

Figure S2. Immunoblot analysis of APOL6 protein amount in MIA PaCa-2 cells transfected with FLAG-tagged APOL6 expression plasmids at different doses. FLAG-tagged APOL6 expression plasmids were transfected at the indicated doses in 6-well plates. The plasmid doses for transfection increased within a particular range (i.e. greater than or equal to 2000 ng), along with increased expression of AOPL6. According to the manufacturer's instructions, the APOL6 plasmid doses for transfection in 96-well plates were set at 100 ng, 150 ng, 200 ng, and 250 ng. The non-specific bands are marked with asterisk (*).

Table S1. Univariate and multivariate analyses of factors associated with response to immunotherapy in the urothelial cancer cohort.

	Statistics	Crude analysis		Adjust analysis	
		OR (95%CI)	*P* value[a]	OR (95%CI)	*P* value[a]
**APOL6 expression**[b]					
Low	153 (65.1%)	1			
High	82 (34.9%)	2.96 (1.65, 5.31)	<0.01	2.08 (0.97, 4.46)	0.06[c]
**Received platinum**					
Yes	185 (78.7%)	1			
No	50 (21.3%)	1.70 (0.88, 3.28)	0.11		
**Sample collected pre-platinum**					
Yes	107 (59.4%)	1			
No	73 (40.6%)	0.69 (0.34, 1.38)	0.29		
**Lund2**					
Basal/SCC-like	48 (20.4%)	1			
Genomically unstable	44 (18.7%)	3.95 (1.63, 9.56)	<0.01		
Infiltrated	58 (24.7%)	0.78 (0.31, 1.95)	0.60		
UroA	73 (31.1%)	0.84 (0.36, 1.98)	0.69		
UroB	12 (5.1%)	1.00 (0.23, 4.31)	1		
**TCGA Subtype**					
I	84 (35.7%)	1			
II	59 (25.1%)	1.47 (0.72, 3.00)	0.30		
III	48 (20.4%)	0.70 (0.30, 1.63)	0.40		
IV	44 (18.7%)	1.24 (0.56, 2.74)	0.60		
**Intravesical BCG**					
No	185 (78.7%)	1			
Yes	50 (21.3%)	1.07 (0.54, 2.11)	0.85		
**Sex**					
Male	186 (79.1%)	1			
Female	49 (20.9%)	0.66 (0.31, 1.37)	0.26		
**Race**					
White	213 (92.2%)	1			
Other	18 (7.8%)	0.96 (0.33, 2.80)	0.94		
**Baseline ECOG Score**					
0	94 (40.0%)	1			
1	131 (55.7%)	0.40 (0.22, 0.72)	<0.01		
2	10 (4.3%)	1.03 (0.27, 3.89)	0.97		
**Tobacco Use History**					
Never	78 (33.2%)	1			
Previous	132 (56.2%)	1.45 (0.78, 2.71)	0.24		
Current	25 (10.6%)	0.55 (0.17, 1.80)	0.36		
**Tissue**					
Bladder	133 (58.1%)	1			

Kidney	46 (20.1%)	0.53 (0.23, 1.19)	0.12
Ureter	32 (14.0%)	0.83 (0.28, 2.49)	0.74
Others	18 (7.9%)	1.13 (0.50, 2.57)	0.76
**Sample age**			
More than 2 years	51 (21.7%)	1	
1-2 years	61 (26.0%)	1.65 (0.73, 3.74)	0.23
Less than 1 year	123 (52.3%)	1.07 (0.51, 2.26)	0.86
**Metastasis Status**			
Liver	68 (32.1%)	1	
Visceral	105 (49.5%)	2.34 (0.99, 5.56)	0.05
Lymph node only	39 (18.4%)	7.12 (2.70, 18.77)	<0.01

[a] *P* values are derived from univariate and multivariate logistic regression models; [b] APOL6 expression: Low and high, respectively, indicate lower than or higher than the mean expression level of APOL6 among the whole urothelial cancer cohort; [c] Adjusted for lund2, baseline ECOG score and metastasis status.

Table S2. Univariate and multivariate analyses of factors associated with overall survival in the urothelial cancer cohort receiving immunotherapy.

	Statistics	Crude analysis		Adjust analysis	
		HR (95%CI)	*P* value[a]	HR (95%CI)	*P* value[a]
**APOL6 expression**[b]					
Low	218 (62.6%)	1		1	
High	130 (37.4%)	0.57 (0.43, 0.75)	<0.01	0.58 (0.42, 0.80)	<0.01[c]
**Received platinum**					
Yes	272 (78.2%)	1			
No	76 (21.8%)	0.71 (0.51, 0.99)	0.05		
**Sample collected pre-platinum**					
Yes	161 (60.5%)	1			
No	105 (39.5%)	1.32 (0.99, 1.76)	0.06		
**Lund 2**					
Basal/SCC-like	66 (19.0%)	1			
Genomically unstable	70 (20.1%)	0.51 (0.33, 0.79)	<0.01		
Infiltrated	92 (26.4%)	0.80 (0.55, 1.15)	0.23		
UroA	102 (29.3%)	0.89 (0.62, 1.28)	0.52		
UroB	18 (5.2%)	1.00 (0.54, 1.84)	0.99		
**TCGA Subtype**					
I	118 (33.9%)	1			
II	95 (27.3%)	0.96 (0.69, 1.35)	0.83		
III	69 (19.8%)	1.23 (0.86, 1.75)	0.26		
IV	66 (19.0%)	0.98 (0.68, 1.42)	0.92		
**Intravesical BCG**					
No	265 (76.2%)	1			
Yes	83 (23.8%)	0.98 (0.73, 1.32)	0.89		
**Sex**					
Male	272 (78.2%)	1			
Female	76 (21.8%)	1.23 (0.91, 1.66)	0.18		
**Race**					
White	318 (92.4%)	1			
Other	26 (7.6%)	0.69 (0.40, 1.18)	0.17		
**Baseline ECOG Score**					
0	134 (38.5%)	1			
1	196 (56.3%)	2.10 (1.58, 2.79)	<0.01		
2	18 (5.2%)	1.97 (1.04, 3.72)	0.04		
**Tobacco Use History**					
Never	116 (33.3%)	1			
Previous	197 (56.6%)	0.90 (0.68, 1.19)	0.44		
Current	35 (10.1%)	1.02 (0.65, 1.61)	0.92		
**Tissue**					

Bladder	195 (57.4%)	1	
Kidney	67 (19.7%)	1.18 (0.85, 1.65)	0.32
Ureter	26 (7.7%)	0.97 (0.59, 1.59)	0.90
Others	52 (15.3%)	0.95 (0.64, 1.40)	0.79
**Sample age**			
More than 2 years	73 (21.0%)	1	
1-2 years	99 (28.4%)	0.78 (0.54, 1.13)	0.19
Less than 1 year	176 (50.6%)	1.09 (0.78, 1.51)	0.62
**Metastasis Status**			
Liver	98 (31.0%)	1	
Visceral	158 (50.0%)	0.61 (0.45, 0.81)	<0.01
Lymph node only	60 (19.0%)	0.35 (0.23, 0.53)	<0.01

[a] *P* values are derived from univariate and multivariate cox proportional hazards regression model; [b] APOL6 expression: Low and High, respectively, indicate lower than or higher than the mean expression level of APOL6 among the whole urothelial cancer cohort; [c]Adjusted for received platinum, lund2, baseline ECOG score and metastasis status.
